# Attitudes and perceptions towards postpartum contraceptive use among seroconcordant partners with HIV in rural Mozambique: a qualitative study

**DOI:** 10.1186/s41256-023-00292-4

**Published:** 2023-03-15

**Authors:** Daniel E. Sack, Almiro Emílio, Erin Graves, Ariano Matino, Paula Paulo, Arifo U. Aboobacar, Caroline De Schacht, Carolyn M. Audet

**Affiliations:** 1 Vanderbilt Institute for Global Health, Vanderbilt University Medical Center, 2525 West End Ave, Suite 750, Nashville, TN 37203 USA; 2 Friends in Global Health, Quelimane, Mozambique; 3 Provincial Health Directorate of Zambézia, Quelimane, Mozambique; 4 Friends in Global Health, Maputo, Mozambique

**Keywords:** HIV, Sexual partners, Contraception

## Abstract

**Background:**

Postpartum contraceptive uptake reduces short interpregnancy intervals, unintended pregnancies, and their negative sequalae: poor maternal and fetal outcomes. Healthy timing and spacing of pregnancy in people living with HIV (PLHIV) also allows time to achieve viral suppression to reduce parent-to-child HIV transmission. There is scant understanding about how couples-based interventions impact postpartum contraceptive uptake among PLHIV in sub-Saharan Africa.

**Methods:**

We interviewed 38 recently pregnant people and 26 of their partners enrolled in the intervention arm of the *Homens para Saúde Mais* (HoPS+) [Men for Health Plus] trial to assess their perceptions of, attitudes towards, and experiences with contraceptive use. Individuals in the HoPS+ intervention arm received joint—as opposed to individual—HIV-related services during pregnancy and postpartum periods, six counseling and skills sessions, and nine sessions with a peer support couple. Our thematic analysis of the 64 in-depth interviews generated 14 deductive codes and 3 inductive codes across themes within the Information, Motivation, and Behavior Model of health behavior change.

**Results:**

Participants reported accurate and inaccurate information about birth spacing and contraceptive methods. They described personal (health, economic, and religious) and social (gender norms, desired number of children) motivations for deciding whether to use contraceptives—with slightly different motivations among pregnant and non-pregnant partners. Finally, they explained the skills needed to overcome barriers to contraceptive use including how engagement in HoPS+ improved their shared decision-making skills and respect amongst partners—which facilitated postpartum contraceptive uptake. There were also several cases where non-pregnant partners unilaterally made family planning decisions despite disagreement from their partner.

**Conclusions:**

These findings suggest that couples-based interventions during pregnancy and post-partum periods aimed at increasing postpartum contraceptive uptake must center pregnant partners’ desires. Specifically, pregnant partners should be allowed to titrate the level of non-pregnant partner involvement in intervention activities to avoid potentially emboldening harmful gender-based intercouple decision-making dynamics.

## Background

Short interpregnancy intervals, which are often the result of unintended pregnancies, can lead to negative maternal and fetal outcomes [1, 2]. Postpartum contraceptive uptake reduces unintended repeat pregnancy and therefore may improve psychosocial, medical, and economic outcomes among people who are able to plan and time their pregnancies [3]. Pregnancy spacing may be particularly important for people living with HIV (PLHIV) who are more likely to experience preterm birth and low birthweight offspring than pregnant people without HIV [4–6]. Appropriate interpregnancy intervals from planned pregnancies, in addition to healthier pregnancies for PLHIV, allows for improved HIV management before/during pregnancy, leading to viral suppression and reduced parent-to-child HIV transmission [5, 7, 8].

This is especially relevant given the high rate of unintended pregnancy among PLHIV in sub-Saharan Africa and the increased parent-to-child HIV transmission during unintended compared to intended pregnancies among PLHIV [9]. Despite the focus on integrating contraception into HIV care in sub-Saharan Africa [10], in 2015 up to 60% of women living with HIV report unmet contraceptive needs [11]—defined as wanting to use, but not having access to, contraception for any reason—and only 7–48% of women of reproductive age in sub-Saharan Africa reported any postpartum contraceptive use [12]. In Mozambique, for example, only 32% of women had their contraceptives needs met in the postpartum period [12]. For pregnant PLHIV, this gap in service delivery leads to a higher risk of parent-to-child HIV transmission [9, 13].

When a pregnant person has HIV, their partner plays a pivotal role in their reproductive choices and engagement in HIV treatment and care [9, 14–20]. In sub-Saharan Africa, strategies that engage partners to improve intra-couple communication have decreased unmet contraceptive needs [21–24]. For example, a 2018 trial found slightly higher postpartum contraceptive uptake (6.4% increased use at eight months postpartum) after educational sessions with both partners during perinatal care [21]. These successes suggest that incorporating interventions that integrate partners into existing maternal health systems/services is a promising avenue for future work.

A review of non-pregnant partner engagement found that, across the 18 interventions assessed, all reported a positive or null association between couples-based interventions and postpartum family planning uptake [25]. A related review of interventions that work within existing gender norms or promote gender equality in reproductive health (contraception, breastfeeding, age at first marriage, etc.) noted either improved or null effects on contraceptive uptake, but highlighted the difficulty in defining causal pathways between gender dynamics and health outcomes [15]. The integration of universal antiretroviral therapy and contraceptive services for postpartum people provides the ideal opportunity to explore drivers of uptake of, and continued support for, contraceptives among a vulnerable population with unmet contraceptive needs.

This study leverages an ongoing cluster-randomized trial—*Homens para Saúde Mais* (HoPS+) [Men for Health Plus] [26]—to gain insight into the key drivers of postpartum contraceptive uptake and choice among PLHIV from the perspective of postpartum people and their partners. Specifically, we used thematic analysis to develop a thematic map within the information, motivation, and behavior model [27, 28], through inductive and deductive coding, that frames perceptions of, attitudes towards, and experiences with contraceptive use among seroconcordant couples living with HIV enrolled in HoPS+ . Finally, we present opportunities for future qualitative and quantitative research questions that will improve postpartum care for PLHIV.

## Methods

### Context and setting

*Homens para Saúde Mais* (HoPS+) [Men for Health Plus] is a clustered randomized controlled trial that explores how incorporating non-pregnant partners into pre- and post-natal care among seroconcordant couples living with HIV influences retention in and adherence to treatment, and parent-to-child transmission in Zambézia Province, Mozambique [26]. Eligible couples included pregnant PLHIV and their non-pregnant partner living with HIV, both at least 18 years of age, presenting for antenatal care services together at one of 24 clinic sites located in Zambézia Province. Both partners must not have been on antiretroviral therapy for 60 days prior to study enrollment—either due to being treatment naïve or lost to follow-up; they must have agreed to start or restart antiretroviral therapy together and receive care services together in the antenatal, postnatal, and/or child-at-risk clinic sectors for themselves and their child; the pregnant partner’s due date must have been greater than two weeks from study enrollment date; and both participants must have been willing and able to provide informed consent for themselves and their children. We only included participants starting or restarting antiretroviral because the intervention required that they be willing to enroll in care together [26].

Participants in the control group received the standard of care prenatal HIV treatment, which included prenatal care and HIV care together for the pregnant partner, with the non-pregnant partner receiving their HIV care separately in the adult HIV services sector. Participants in the intervention group, in addition to joint ante- and post-natal and HIV care, received an intervention package of six couples counseling sessions that included discussions about communication, shared decision making, and conflict resolution, including how these topics relate to contraception, as well as education about HIV. Participating couples in the intervention group were also paired with an expert peer support couple from the same community to provide them with additional peer counseling, guidance, and encouragement through nine monthly community- or home-based visits during the antenatal and postpartum periods [26]. The HoPS+ trial also collected postpartum contraceptive data and therefore provides an opportunity to gain insight into how engaging non-pregnant partners in prenatal care influences postpartum contraceptive uptake among PLHIV in Zambézia Province, Mozambique.

Zambézia Province is a mostly rural province in north-central Mozambique [29]. It has some of the lowest health and development indicators in Mozambique [30]. While HIV prevalence in Mozambique was 12.5%, approximately 17.1% of adults in Zambézia were living with HIV in 2021 [31]. Among PLHIV in Zambézia in 2021, an estimated 97.1% were on treatment and 91.8% of people on treatment were virally suppressed, up from 30 and 50% respectively in 2015 [31, 32]. The Mozambican Ministry of Health provided the following contraceptive methods free of charge at all maternity wards, immediately postpartum, and at any postpartum care visits: combined contraceptive pills, progesterone-only pills, injectables (both intramuscular and subcutaneous), copper intrauterine devices, implants, tubal ligation (female sterilization), vasectomy (male sterilization), condoms (male and female), and an emergency contraceptive pill (personal communication, 2021). As of Family Planning 2020 estimates, approximately 31% of women were using contraception in 2022 [33].

### Study design

This qualitative component of the HoPS+ trial included participants recruited from six of the 12 intervention sites to participate in in-depth qualitative interviews between 12 and 18 months after study enrollment. Interviews assessed each individual’s experience with the intervention activities, healthcare costs-related to their healthcare services use, intervention fidelity and acceptability, and suggested improvements to couple-based services in the health facility and community. Interviews with 25 female and 25 male participants from six different clinics would ensure data saturation for contraceptive topics in this population [34]. However, more interviews were planned as part of the HoPS+ trial due to the greater data saturation needs for other planned analyses and we analyzed the interviews after they were completed due to the delay from transcribing and translating the interviews. Specifically, couples enrolled in the full HoPS+ trial at six of the 12 intervention sites were randomly selected using a randomly generated priority sampling list until 3–5 couples were selected from three of the intervention sites with the largest patient population and three of the intervention sites with the smallest patient population. This resulted in in-depth qualitative information from 38 female participants and 26 male participants enrolled at six different clinic sites (64 total), despite reaching data saturation after analyzing 50 of the transcribed interviews.

### Interview development

Prior to the start of the interview process, we created three qualitative questions aimed to elicit perceptions of, attitudes towards, and experiences with contraceptive initiation to supplement the HoPS+ questions (Table 1). Specifically, we developed a thematic map within the Information, Motivation, and Behavior Model [27, 28], through inductive and deductive coding, that framed perceptions of, attitudes towards, and experiences with modern contraceptive use among seroconcordant couples living with HIV in HoPS+ . We also looked to the situated-information, motivation, and behavior model for insight into how to include intrapersonal and social codes that may influence postpartum contraceptive uptake [35]. Questions (Table 1) elicited perceptions of (information), attitudes towards (motivation), and experiences with (behavior) modern contraception among participants. We employed a thematic approach to identify and analyze themes in the data after repeated reading (Table 2), using a combination of inductive coding and deductive codes based on findings from published literature [36, 37].

**Table 1:** Qualitative interview guide

Number	Question
1)	**How many children do most couples have here in your district (or zone)? What are the more frequent reasons to have that number of children?**
2)	How many children would you like to have?
3)	**Was your last pregnancy/child planned (between you and your partner)?**
4)	**Did you and your partner talk about using contraceptives/ any type of family planning?**
	**Tell me about any discussions you and your partner had about using contraceptives/family planning?**
	How, if at all, did your counseling sessions in this study help you and your partner discuss using contraceptives/family planning?
5)	Would you like to have more children?
	**If yes, when would you like to have your next child?**
	**If no, are you currently doing something to prevent pregnancy in your relationship?**

Boldface indicates an updated question after the COVID-19-induced pause in interviews

**Table 2:** Codebook

Theme	Code (type)	Definition	Examples
Contraceptive knowledge	Accurate information (deductive)	Expressed factually correct information about contraceptive methods (or where to get it) and pregnancy	"It helped because he helped, right, he explained to me, I went and advised my husband, and he accepted that actually the children are little. If you don’t you won’t be in good health, you should rest and let the children grow up, then let’s have another child later." (20-year-old female, Inhassunge) "They told us we can use a condom when we have sex outside, yes." (39-year-old male, Gilé)
	Misinformation (deductive)	Expressed factually incorrect information about contraceptive methods (or not knowing where to get it) and pregnancy	"No, she’s only a year and three months, she’s still very little for her mother to get pregnant again. And she is still breastfeeding, it will only be when she weans from breastfeeding….We haven’t weaned her so this helps the mother avoid pregnancy, and also, right, right we didn’t start having sexual intercourse because the baby is still breastfeeding" (27-year-old male, Mocubela) "I usually see women doing planning while the children are still in their belly, if they don’t comply with health’s law they will die.” (38-year-old male, Pebane)
	New information (deductive)	Any reference to new information that either member of the couples learned from any of the HoPS+ sessions about contraception, birth spacing, or reproduction	"It’s because where we come from we didn’t know there were pills you could take to decrease the number of children. So now that there is medication, if you want to have 2 or 3 then you just go to the hospital. When you go to the hospital they give you medication so those children can grow up. That’s what I think now." (36-year-old male, Quelimane) "Helped me by saying that you need to give good medication for the child, medication for you too, so that you’re well. If you don’t give medication to the child, and if you don’t take it the child will also not have good health. I memorized that…They said that in order to having children you need… if you see that you already gave birth and the child is already 2 years old and I don’t give it a break, you ought to go to the hospital to ask for medication and wait for the child to grow up a little. After you see the child is at a good age, you can have another, you can stop [family] planning to have another." (20-year-old female, Inhassunge)
Partner motivation	Individual fertility desires (deductive)	Rationale for why an individual wants children (take care of them when they are old, child mortality, financial difficulties with many children, etc.) or why they want to delay or stop having children	"I would like to rest first." (20-year-old female, Mocubela) "I want to have a maximum of four (4) because if you give birth to many is not a bad thing to get old, right?" (28-year-old male, Maganja Da Costa) "May be twenty I would like (smiles), because it’s good to have children at home." (20-year-old female, Gilé) "The reason I want ten children, for example, my mother only had me, only me alone, yes, the brothers all died, the mother died, and just left with dad. So is the reason I ask and say at least ten." (39-year-old male, Gilé) "They [the children] can take care of each other, welcome each other." (19-year-old female, Pebane)
	Health considerations (inductive)	Expressing that improved health has changed how an individual or partners think about having additional children or that health limits future fertility goals	"If I feel like I have good health, maybe I will think about having more children." (23-year-old male, Mocubela) "My health was very weak before taking the medication. It was health hour by hour. Now I see that it is different from what it was before. Now I go a month without feeling sick." (20-year-old female, Inhassunge)
	Economic considerations (deductive)	Any reference to economic considerations in making fertility decisions	"It could be four… but even having four in the times we’re living in, I don’t know, if it was old times where you had a lot of food for children to eat, people would have 7 even 10…Now, the way I see it, these days if someone has 4 or 3 it is already too much." (27-year-old female, Inhassunge) "I used to think I could have a maximum of 3 or 4. Because here in Mozambique, to have 9 or 10 children, ehhh, poverty will add to poverty, hmm." (28-year-old male, Gilé)
	Religiosity (deductive)	How an individual considers religious preferences in fertility decisions	"Those [children] who God wants to give us, according to his will." (19-year-old female, Gilé) "Since our religion says that planning is a sin, that’s why I say that it will depend on the number God wants to give me. Even if it’s 1 we will thank this God who gave us 2, 3… hm." (27-year-old male, Mocubela)
	Social norms/motivations (deductive)	Expressed social norms surrounding fertility in couples' community	"There are people who gave birth to 10 and people they say gave birth too much. Some give birth to 5 and they also say it was too much. There are people who birthed 4, 2 girls and 2 boys and they say that one birthed well…Girl pounds flour, fetches water. Boy will also take you to the hospital when you’re sick, talks to the nurses, that’s what a boy does. So you don’t say it’s not good to have a boy." (20-year-old female, Mocubela) "It depends on each person’s wishes. They agree between the couple, the husband along with his wife will say, “I want these many children”, the women will give birth until they reach the number they agreed on. Afterwards you just need to do family planning." (27-year-old male, Mocubela)
Barriers to contraceptive uptake	Paternalism (deductive)	If the female partner reports that the male partner wants control family planning or the male partner reports wanting to control family planning	"Yes, she has been saying that “when the day comes for me to go do (family) planning, I will go to do (family) planning”. So I told her that she can’t do planning alone without discussing with me. We should agree first and then do the planning. That’s all.” (19-year-old male, Quelimane)
	Gender norms (deductive)	Examples of partners acting in ways that would be expected of them given their gender	"I don’t know [how many children I would like] since my husband isn’t here." (23-year-old female, Maganja Da Costa)
	Surreptitious contraceptive use (deductive)	References to hiding contraceptive use from partner (e.g., reporting using injectables so partner does not know)	"We didn’t agree [on using family planning]. She did it and when I found out about the idea, I liked it. She said she wanted to rest. “I don’t want to have another child while this one is little,” I said alright." (22-year-old male, Mocubela) "When I talk he won’t listen. So I want to try to “steal” by myself." (30-year-old female, Inhassunge)
Facilitators of contraceptive uptake	HoPS+ Engagement (deductive)	References to partner’s engagement to HoPS+ intervention components, includes attendance at counseling/skills sessions or peer sessions as well as references to what a partner shared at or after any sessions	"It helped because he [the couples counselor] helped, right, he explained to me, I went and advised my husband, and he accepted that actually the children are little. If you don’t you won’t be in good health, you should rest and let the children grow up, then let’s have another child later." (20-year-old female, Inhassunge) "Yes, my relationship with my wife really did change. I can tell you how it changed, it changed a lot because we didn’t understand each other before, we would each accuse the other. So then this phase arrived, we are established, no one accuses the other, everything is normal, it’s normal. We live without any problems, no arguments, we are able to talk and get over problems." (34-year-old male, Maganja Da Costa)
	Trust in providers (deductive)	Expressed trust in providers, counselors, or peer support couples to give advice/information on contraceptive decisions	"When they arrived and gave me advice, and explained everything to me, I was free to feel that emotion, with that happiness. Yes, I like those people that were coming to the house, yes, I can’t speak ill of them." (39-year-old male, Gilé)
	Partner respect (deductive)	Expressed respect or examples of listening to or following partner's desires (or, conversely, not doing so)	"We help each other. Sometimes when I’m sick, or even my son, or I went to another place, I say, “husband go get mine for me,” and if it was him, he also says, “wife go get it for me too and bring it home.” We haven’t argued at our home yet…It helped us because when we get home we respect each other as husband and wife, we don’t fight because of this disease." (20-year-old female, Gilé)
	Shared decision making (inductive)	References to talking to partner (or avoiding talking to partner) about using contraception	"It depends on each person’s wishes. They agree between the couple, the husband along with his wife will say, “I want these many children”, the women will give birth until they reach the number they agreed on. Afterwards you just need to do family planning." (27-year-old male, Mocubela) "We have said that, “oh my friend, we have to let this child grow a little. We do the planning and when it grows up, we leave the planning.”” (19-year-old female, Quelimane)
	Paternalism (deductive)	If the female partner reports that the male partner wants control family planning or the male partner reports wanting to control family planning	“Yes… my husband forced me [to have an implant]…He’s the one who forced me." (18-year-old female, Maganja Da Costa)
Postpartum contraceptive use	Current contraceptive use (inductive)	Reported current contraceptive use in female partner (or by male partner)	"I put in an implant." (18-year-old female, Maganja Da Costa) "She’s using contraceptives, it’s an implant.” (23-year-old male, Maganja Da Costa)

**Fig. 1:**
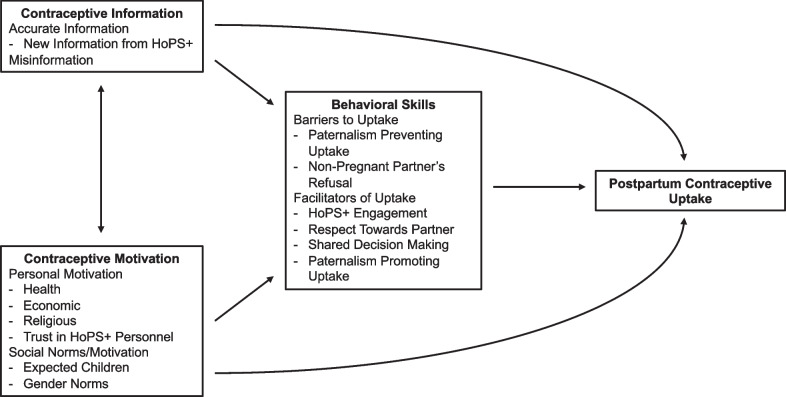
Framework of modern postpartum contraceptive uptake

Questions assessed how participant preferences were (or were not) communicated postpartum and important considerations that potentially influenced contraceptive uptake. They also elicited how the HoPS+ intervention, targeting shared decision making and communication skills, impacted couples’ contraceptive decision-making process. We used existing literature to predict themes and compared what the HoPS+ trial participants expressed to the perceptions and attitudes presented in literature that similarly assessed participants’ perceptions and attitudes towards modern contraception in Mozambique and sub-Saharan Africa [15, 16, 18, 24, 38–42]. These data allowed us to supplement and modify the theoretical model guiding the study intervention—the Information, Motivation, and Behavior model and its situated adaptation [27, 28, 35]—on postpartum contraceptive uptake in Mozambique and sub-Saharan Africa (Fig. 1). The Information, Motivation, and Behavior Model posits that risk-reduction behavior, risk-reduction motivation, and behavioral skills, which are mutually reinforcing, drive risk-reduction behaviors and lead to favorable outcomes [27, 28, 35].

The first 29 interviews were completed before the COVID-19 pandemic in Mozambique led to a pause in interviews and other select study intervention activities. Based on these interviews, we updated the questions to elicit more comprehensive information about perceptions of, attitudes towards, and experiences with modern contraceptive use among couples living with HIV enrolled in HoPS+ intervention group. The updated questions are presented in boldface in Table 1. Specifically, three questions with additional follow-up probes were added to the existing questions. We interviewed an additional 35 participants with the updated question list after the COVID-19-induced pause.

### Data collection and processing

Trained study personnel conducted in-depth qualitative interviews with each participant separately. Interviews occurred in a private space at the health facility, the couples’ home, or another location agreed upon by the participant and study staff. Interviews were administered in the participants’ preferred language. Two bilingual study personnel translated responses into Portuguese separately and iteratively checked each other’s translations until they reached consensus. A third bilingual person mediated any disagreements. This process was repeated from Portuguese to English for the questions in Table 1.

### Data analysis

Authors DES and CMA generated codes and highlighted themes by collating codes across the data set and reviewing themes to develop a thematic map (e.g., the relationship between relationship support and uptake of contraceptives) (Table 2, Fig. 1). We identified 14 deductive codes and 3 inductive codes across three themes [15, 16, 18, 24, 38–42]. We used MAXQDA2020^®^ software to ensure consistency across coding and analysis. The final coding framework had at least 85% agreement between two coders without the need for further discussion after one meeting—although we did discuss divergent codes and mutually agreed on the best final option. We found a *Contraceptive Information* theme that included accurate and inaccurate contraceptive information, a *Contraceptive Motivation* theme, which included both personal and social motivations, and a *Behavioral Skills* theme, which included skills that either facilitated or were barriers to modern postpartum contraceptive uptake in the Information, Motivation, and Behavior Model framework (Fig. 1).

## Results

### Demographic characteristics

The baseline demographic, psychometric, and clinical characteristics of the 64 interviewed participants are presented in Table 3. Female participants were evenly recruited before and after the COVID-19-induced interview pause, whereas more male participants were recruited afterwards (42.3% before vs 57.7% after). Female interviewees were younger (median age 22.5 years, interquartile range [IQR] 20–26.8 years) than male interviewees (median age 27.5 years, IQR 25.3–31.5 years); and more likely than males to describe their relationship status as “single” (50% vs 34.6%), with very few interviewees of either sex describing their relationship status as “married”. Female interviewees had less formal education (in years completed) than males and were more likely to work as farmers (60.5% vs 30.8%). Female and male interviewees had very similar median scores across all psychometric instruments. Females, however, attended a greater percentage of couples’ counseling and skills sessions (median 100%, IQR 83.3–100%) and peer support sessions (88.9%, IQR 77.8–100%) compared to males (66.7%, IQR 50–83.3% and 66.7%, IQR 55.6–86.1%, respectively).

**Table 3:** Demographic, psychometric, and clinical data among qualitative interviewees, collected at HoPS+ enrollment

	Female	Male
	(n = 38)	(n = 26)
*Enrollment year*		
2018	15 (39.5%)	9 (34.6%)
2019	5 (13.2%)	4 (15.4%)
2020	18 (47.4%)	13 (50.0%)
Recruitment relative to COVID-19 pause*		
Before	18 (47.4%)	11 (42.3%)
Afterwards	20 (52.6%)	15 (57.7%)
*Age (years)*		
Median [Q1, Q3]	22.5 [20.0, 26.8]	27.5 [25.3, 31.5]
*District*		
Pebane	5 (13.2%)	4 (15.4%)
Inhassunge	7 (18.4%)	3 (11.5%)
Mocubela	9 (23.7%)	7 (26.9%)
Maganja da Costa	10 (26.3%)	5 (19.2%)
Namacurra, Gilé, or Quelimane	7 (18.4%)	7 (26.9%)
*Relationship status*		
Living together	18 (47.4%)	16 (61.5%)
Single	19 (50.0%)	9 (34.6%)
Married	1 (2.6%)	1 (3.8%)
*Education*		
None	4 (10.5%)	0 (0%)
Some primary school (grades 1–7)	28 (73.7%)	13 (50.0%)
More than some primary school	6 (15.8%)	13 (50.0%)
*Occupation*		
Farmer	23 (60.5%)	8 (30.8%)
Domestic	14 (36.8%)	–
Fisher	0 (0%)	9 (34.6%)
Other	1 (2.6%)	9 (34.6%)
*Perceived community stigma*		
Median [Q1, Q3]	15.0 [8.50, 23.5]	16.0 [10.0, 29.0]
Missing	14 (36.8%)	7 (26.9%)
*Patient felt/experienced stigma*		
Median [Q1, Q3]	13.0 [9.00, 14.0]	12.0 [11.0, 15.0]
Missing	9 (23.7%)	4 (15.4%)
*Physician trust*		
Median [Q1, Q3]	35.5 [32.0, 39.0]	35.5 [33.8, 39.0]
Missing	10 (26.3%)	6 (23.1%)
*Cognitive empathy*		
Median [Q1, Q3]	24.0 [15.0, 25.0]	20.0 [15.0, 24.0]
Missing	19 (50.0%)	7 (26.9%)
*Affective empathy*		
Median [Q1, Q3]	12.0 [8.50, 13.0]	11.0 [6.50, 12.0]
Missing	16 (42.1%)	10 (38.5%)
*Perceived support*		
Median [Q1, Q3]	25.5 [21.0, 28.0]	25.5 [23.0, 28.0]
Missing	4 (10.5%)	2 (7.7%)
*Needed support*		
Median [Q1, Q3]	28.5 [24.0, 32.0]	30.5 [24.8, 32.0]
Missing	8 (21.1%)	2 (7.7%)
*HIV knowledge (0–27)*		
Median [Q1, Q3]	19.5 [17.0, 21.0]	18.0 [14.5, 19.5]
Missing	18 (47.4%)	11 (42.3%)
*Patient health questionaire-9*		
Median [Q1, Q3]	2.00 [0, 7.50]	1.50 [0, 3.25]
Missing	7 (18.4%)	6 (23.1%)
*Proportion of skills sessions attended*		
Median [Q1, Q3]	1.00 [0.833, 1.00]	0.667 [0.500, 0.833]
*Proportion of peer sessions attended*		
Median [Q1, Q3]	0.869 [0.778, 1.00]	0.667 [0.556, 0.861]
*Using modern postpartum contraception*		
Yes	14 (36.8%)	–
No	14 (36.8%)	–
Missing	10 (26.3%)	–

*Q1* first quartile; *Q3* third quartile

*Interviews were paused in March 2022 and resumed in September 2022 due to the COVID-19 pandemic

While not all participants answered questions about their current number of children or desired number of children in the in-depth interviews, answers ranged from zero to six current children and anywhere from three desired children to as many children as possible (with as high as 20 children for one participant), with most participants hoping for four children in total. Below, we explore participant responses to each theme depicted in Fig. 1 with each code defined in detail in Table 2. To provide context to each participant quote, we label it with their age, sex, and district.

### Contraceptive information

Contraceptive information encompassed accurate information, including new information about contraception gleaned from participating in the HoPS+ intervention activities, and misinformation.

Several participants shared the importance of birth spacing to prevent deleterious maternal and infant outcomes, with one explaining:

*It helped because [the HoPS*+ *counselor] explained to me [about birth spacing], I went and advised my husband, and he accepted that actually the children are little. If you don’t [wait] you won’t be in good health, you should rest and let the children grow up, then let’s have another child later* (20-year-old female, Inhassunge).

Other participants applied this knowledge to practical developmental milestones, with one explaining, “After [the child is] walking, [you can] stop the [family] planning” (24-year-old male, Mocubela).

While participants frequently shared information about birth spacing—some even described plans to only have another child in a set number of years—very few shared any information they learned about different family planning options. One male participant expressed that, “we didn’t know there were pills you could take to decrease the number of children” (36-year-old male, Quelimane). There was no substantive discussion, even when prompted, about which type of contraception was best or preferred.

While some participants shared information about the lactational amenorrhea method (LAM), not all of it was strictly accurate. For example, while one participant correctly explained that “We haven’t weaned her so this helps the mother avoid pregnancy” (27-year-old male, Mocubela), however, he later reveals that the infant is 15 months old, beyond the timepoint at which LAM is considered highly efficacious for preventing pregnancy [43, 44].

### Contraceptive motivation

Contraceptive motivation encompassed participants’ personal motivation, including how health, economic, and religious considerations influenced fertility and/or family size desires, and newfound trust in HoPS+ study personnel, and participants’ views of the social norms and motivators influencing fertility/reproductive choices in the region.

In general, female participants expressed a desire to, as one bluntly put it, “rest first” (30-year-old female, Inhassunge) prior to subsequent pregnancies. Other female participants commented on the physical stressors that accompany pregnancy, with one explaining, “I suffer a lot when I get pregnant” (26-year-old female, Namacurra), and another agreeing:

*When I get pregnant my heart starts hurting…I can’t do any house chores; when I realize I’m pregnant I don’t do anything at home, I can’t because my heart starts hurting* (22-year-old female, Mocubela).

Beyond the physical, some female interviewees reported other practical barriers to subsequent pregnancies, such as not having family members to help, or needing their current children to be old enough to help with subsequent children (hence the desire to hold off for now). One female participant explained:

*It’s a thought I’ve had for a long time because when you give birth to another child before the first is grown up, it’s going to be your own suffering. Because a person will carry one [child] on their back as well as the pregnancy, then it’s suffering. Now if one [child] is bigger, it will carry the other, and you as the mother will carry the bundle [of belongings]* (38-year-old female, Mocubela).

Several participants were also very cognizant of their economic situation and how it impacts their fertility desires. One male participant explained, “I used to think I could have a maximum of 3 or 4 [children]. Because here in Mozambique, to have 9 or 10 children, poverty will add to poverty” (28-year-old male, Gilé). While some participants agreed, such as a female participant who said, “even having four in the [difficult economic] times we’re living in, I don’t know…” (27-year-old female, Inhassunge), other participants disagreed, suggesting that additional children could help with wealth generation in a family. One male participant elaborated, with a caveat:

*…all children can’t be poor. Even the poor one will come to his Dad’s house when Dad is not well and cut wood, cut grass, that’s the advantage of having a lot of children. But you need to be able to raise them* (38-year-old male, Pebane).

Many participants also reported that their religious beliefs impacted their family planning choices. Several made statements like that of one female participant, who when asked how many children she would like, responded, “Those who God wants to give us, according to his will” (19-year-old female, Mocubela). A male participant elaborated that, “Since our religion says that [family] planning is a sin, that’s why I say that [the number of children] will depend on the number God wants to give me” (27-year-old male, Mocubela).

Male participants were more likely to express interest in having a relatively larger family. One male participant shared his reference point from his previous lived experience:

*The reason I want ten children, for example, my mother only had me, only me alone, yes, the brothers all died, the mother died, and just left with dad. So is the reason I ask and say at least ten* (39-year-old male, Gilé).

Another male interviewee focused on what children could provide him later in life, explaining, “When someone has 6 children, if there’s no bad luck, you might get a few that will help you when they’re grown up” (23-year-old male, Pebane). A few participants also reported that their newfound good health, after starting antiretroviral therapy for their HIV, stimulated thoughts about having additional children. One male participant summarized, “I feel like I have good health, maybe I will think about having more children” (23-year-old male, Mocubela).

Participants reported that the HoPS+ study personnel generated a great degree of trust, which facilitated both antiretroviral therapy adherence and an openness to using postpartum contraceptives. One female participant summarized, “If they [the HoPS+ peer support couple] come to my house and tell me something, I will also do that because they told me to” (25-year-old female, Gilé).

Participants reported that social norms encouraged couples to have as many children as they wanted. There were, however, social expectations for children’s contribution to the household tasks. A female participant explained, “Girls pound flour [and] fetch water. Boys will also take you to the hospital when you’re sick [and] talk to the nurses” (20-year-old female, Mocubela). Another female participant elaborated:

*Because when the children are grown they can help you...When they are men and women: some go to the river, others gather firewood, and if they’re grown they’ll help their mother* (25-year-old female, Pebane).

Female participants’ frequent inability to respond to the question about the number of children they desired highlighted a strong social norm for women to defer to their male partners for such decisions. One female participant refused to answer the question, noting “I don’t know [how many children I want], since my husband isn’t here” (23-year-old female, Maganja da Costa). Another had a similar response, explaining “I don’t know because he’s not home…he would be able to tell me. But my wish alone is that I would like to rest [from having more]” (27-year-old female, Inhassunge). This deferment to one’s male partner aligned with the trend seen across all interviews that, in general, male participants were more expressive about their contraceptive attitudes and attitudes towards additional children than female participants.

Perceived stigma surrounding pregnancy and childbirth for those living with HIV appears to have decreased substantially with the introduction of antiretroviral therapy and the resulting improvements in participant health and reduction in the risk of parent-to-child HIV transmission. One male participant clarified:

*…[starting treatment] changed a lot, I mean that now I’m making children that are not HIV positive, so this has changed a lot for me. Another thing, health itself. When the community sees us it’s a little different [than before we had treatment]: ohhh that couple is not like before. But, through them [the peer couple], with the explanation they bring us here at home, they are making us change a lot. For example, society, or at home, family too, they all started seeing that ohh our children are living well, they’re living more. So it’s more to do with health… its normal to live a healthy life* (34-year-old male, Maganja da Costa).

### Behavioral skills

Participants reported skills they used to overcome barriers to contraceptive uptake, such as the need for surreptitious contraceptive use, and skills gained, primarily though engagement in HoPS+ , such as showing respect to their partner, shared decision making, and how to navigate paternalism, to facilitate contraceptive uptake.

Several male participants noted, for example, that they should be in control of their female partners’ fertility/reproductive decisions moving forwards. In this scenario, if the male partner refused to support contraceptive use, the woman would be left with two options: choose to take/use contraceptive(s) clandestinely (e.g., via an injectable contraceptive method) or risk a possible unwanted pregnancy. One male participant explained, “I told her that she can’t do [family] planning alone without discussing with me” (19-year-old male, Quelimane).

One female participant, who reported using injectable contraceptives to the HoPS+ study interviewer, felt the need to hide her contraceptive use from her male partner, explaining, “When I talk he won’t listen. So I want to try to ‘steal’ [take contraceptives] by myself” (30-year-old female, Inhassunge). A male partner also expressed surprise when his partner started contraception without his input. He, however, was open to it, reporting, “She did it and when I found out about the idea, I liked it. She said she wanted to rest…I said alright” (22-year-old male, Mocubela).

Most participants reported good engagement with HoPS+ activities and personnel. Engagement with the HoPS+ intervention also facilitated respect and shared decision making among participants. Both the female and male participant from the same couple (26-year-old female & 26-year-old male, Namacurra) separately agreed that they engaged in shared decision making to reach a “consensus” about family planning. Another participant described how the HoPS+ couples counselor encouraged him to engage in shared decision making with his partner (and provided an example of them doing so):

*When we went to the hospital, she [the couples counselor] told us about [family] planning, and she [the couples counselor] said, “go home, talk to each other and when you come back next time tell me what you think about the subject.” We talked, so when it’s time we are going to do it [family planning]* (27-year-old male, Mocubela).

While participants frequently reported that paternalism could lead women to forgo contraceptives, several participants reported examples where it had the opposite effect. In one case, a male participant reported, “we [my wife and I] talked about [family planning] first and then we made this decision [to use it]” (23-year-old male, Maganja da Costa). He was later contradicted by his partner, who admitted that her “husband forced me [to have an implant] …he’s the one who forced me” (18-year-old female, Maganja da Costa).

## Discussion

The 64 interviewed HoPS+ participants’ perspectives, particularly with the inclusion of male partners, add to the literature on contributors to postpartum contraceptive uptake among pregnant PLHIV. For example, Agadjanian and Hayford [40] postulated that improved access to antiretroviral therapy in southern Mozambique tempered a “now-or-never” approach to childrearing in rural Mozambique among PLHIV [40, 45]. Specifically, PLHIV in the region had previously worried that, given their (perceived) impending death prior to the widespread availability of antiretroviral therapy, they needed to have as many children as possible as soon as possible (or not have any children) [45]. Female and male participants in this study expressed support for this attitude shift towards more planned and spaced subsequent pregnancies in the context of treatment availability. In addition to highlighting their own improved health on antiretroviral therapy, they were optimistic about preventing HIV acquisition among their children. Additionally, there is evidence that religious beliefs are essential contributors to family planning decisions in Mozambique [39], which was also evident in these interviews among both female and male HoPS+ participants.

Participants of both sexes also supported the principle of “resting” between pregnancies to make sure they and their families are healthy and/or prepared for subsequent pregnancies. This aligns with the principles of “healthy timing and spacing of pregnancies”, a family planning approach which aims to promote 24 months between pregnancies, per 2005 recommendations from the World Health Organization [4], in programs that provide reproductive health services [46]. The idea of healthy timing and spacing was implemented across United States (US) government-funded programs starting in the 1990s, including the President’s Emergency Plan for AIDS Relief (PEPFAR) in the 2000s, because it was explicitly exempted from the Mexico City Policy—which originally prevented foreign organizations from using US government funding to perform or promote abortion services and, under President Trump, was expanded to restrict the use of any development programs, PEPFAR included, for the same purpose [46, 47]. This is highly relevant given that, since the early 2000s, the US has given more official development assistance than other high income countries (albeit, a lower share of their gross domestic product is directed towards development assistance) [48]. While we do not have data from before healthy timing and spacing’s widespread incorporation into global reproductive health programming, the HoPS+ participants frequently acknowledged the importance of birth spacing, which suggests that the core messaging—on the health benefits of birth spacing—has been rather effective. However, these data also raise pressing questions about intervention planning and contraceptive knowledge that require further examination.

Specifically, this study elevates the important question of how to include pregnant and non-pregnant partners when intervening on contraceptive uptake, particularly given the gendered perspectives evident in this analysis. This aligns with previous work in Mozambique suggesting that female and male partners play different roles in contraceptive decision-making [17]. Other data indicates that improved spousal communication and improved shared decision making skills, such as those gained from HoPS+ intervention sessions, may positively influence contraceptive uptake in sub-Saharan Africa [16, 41]. Importantly, however, we also found cases where pregnant partners were forced into using postpartum contraceptives, felt the need to use contraceptives without their partners’ knowledge, or reported that their non-pregnant partners made reproductive decisions for them. This suggests that communication and shared decision making strategies remain limited, particularly in cases with male partners who range from disinterested to unsupportive to abusive, which may inadvertently decrease access to care and lead to worse pregnancy outcomes [49]. This complicates intervention planning for pregnant people with partners intent on controlling their reproductive decision making [50]. Interventions should therefore center pregnant partners and allow them to titrate the level of non-pregnant partner involvement based on their personal needs.

Finally, while we had hoped the interview questions would elicit participants’ reasons for using particular contraceptive methods, participants did not provide detailed information on why they were using (or preferred) one method over another—although contraceptive availability may have played a role—outside of their use of LAM via breastfeeding. Consensus guidelines on LAM, which suggest high efficacy (98%) at preventing pregnancy for the first six months of the postpartum period, however, require exclusive breastfeeding, amenorrhea, and an infant less than six months old [44]. Further information on other methods would help guide future interventions aimed at increasing highly efficacious contraceptive uptake after six months postpartum. For example, some scholars have posited that injectable contraceptives are popular because they allow people seeking to prevent pregnancy to use a highly effective contraceptive method without the knowledge of their partner or family members [51]. They could also be popular because they only require a trip to the clinic for an injection every three months (which also is convenient for new parents taking their children for immunizations) rather than a daily pill or a device insertion [51]. Given that there is evidence that male partners make the reproductive health decisions in Mozambique for over a quarter of couples [17], which some HoPS+ participants also suggested, more information on why individuals select specific contraceptive methods would be helpful and would require additional qualitative studies.

These results are also subject to several additional methodological limitations. HoPS+ participant perspectives may not be transferable to more urban regions of Mozambique (or elsewhere in sub-Saharan Africa) due to different life experiences, fertility or reproductive priorities, and religious beliefs in different regions. Additionally, by design, these interviews did not include individuals who did not attend HoPS+ intervention sessions. As such, these interviews did not provide insight into how to improve engagement in future behavioral interventions aimed at increasing postpartum contraceptive uptake. Furthermore, for the most part, participants did not provide examples of how the HoPS+ intervention or the health system increased or decreased contraceptive uptake, which would have provided additional information to guide future interventions. Finally, we were not able to elicit participants’ insights into how other structural barriers to contraceptive use (e.g., transportation, time constraints, etc.) may impact whether they chose to use contraceptives in the postpartum period.

## Conclusions

This relatively large qualitative study in a rural population, with female and male partners represented, reinforces existing data on postpartum contraceptive use and provides useful insight for future research. Specifically, participants affirmed that the “healthy timing and spacing of pregnancies” campaign’s message resonates among both pregnant and non-pregnant people in Zambézia province. They also highlighted the influence of religion, relationship dynamics, and non-pregnant partners on postpartum people’s reproductive decisions. These data further suggest that future interventions will have to the strike the right balance between promoting agency (in both non-pregnant partner involvement and reproductive and family planning decisions) and creating broadly applicable programs that do not impose a one-size fits all approach to the detriment of some participants. These programs cannot overlook and must be cognizant of how contraceptive methods have been used to promote white supremacy, colonialism, and sexism, particularly in Southern Africa.

## Data Availability

The datasets used and/or analyzed during the current study are available from the corresponding author on reasonable request. The codebook is available as a table in this manuscript.

## Abbreviations

COVID-19: Coronavirus disease 2019
HIV: Human immunodeficiency virus
HoPS+: *Homens para Saúde Mais* [Men for health plus]
IQR: Interquartile range
LAM: Lactational amenorrhea method
PEPFAR: President’s emergency plan for AIDS relief
PLHIV: People living with HIV
